# On the equivalence between non-factorizable mixed-strategy classical games and quantum games

**DOI:** 10.1098/rsos.150477

**Published:** 2016-01-27

**Authors:** Azhar Iqbal, James M. Chappell, Derek Abbott

**Affiliations:** School of Electrical and Electronic Engineering, The University of Adelaide, Adelaide, South Australia 5005, Australia

**Keywords:** quantum games, game theory, quantum probability

## Abstract

A game-theoretic setting provides a mathematical basis for analysis of strategic interaction among competing agents and provides insights into both classical and quantum decision theory and questions of strategic choice. An outstanding mathematical question is to understand the conditions under which a classical game-theoretic setting can be transformed to a quantum game, and under which conditions there is an equivalence. In this paper, we consider quantum games as those that allow non-factorizable probabilities. We discuss two approaches for obtaining a non-factorizable game and study the outcome of such games. We demonstrate how the standard version of a quantum game can be analysed as a non-factorizable game and determine the limitations of our approach.

## 1. Introduction

The realization has now emerged [1–3] that the processing of information cannot be separated from the underlying fundamental physics and that the physical aspects of information processing must be taken into consideration. This has led to a new understanding of the information processing, cryptography, and the methods and techniques used for communication that are based on the rules of quantum theory [4].

A venue where information plays a central role is in the established branch of mathematics called game theory [5–7] that provides the necessary mathematical tools, methods and solution concepts for the analysis of conflict. The understanding of games is based on some fundamental assumptions relating to what information is available to each participating player at a particular stage of the game. The finding that physical aspects can have a crucial role in information processing has natural implications for player strategies, and the considerations of underlying fundamental physics become important for game theory. Such questions have led to creation of the research area of quantum games [8–70]. What these studies have shown is that quantum strategies can result in outcomes that often defy our classical intuition.

The emergent field of quantum game theory is rapidly growing [71]. Quantum game theory has two branches: (i) games based on quantum coin tossing that explore the theory of quantum walks, and (ii) strategic games, in the von Neumann sense, which explore quantum decision spaces in situations of conflict. There are a number of directions that have motivated research in quantum game theory. First and foremost, it must be recognized that the field is a fundamental exploration of scientific curiosity and that it provides a glimpse into quantum dynamics. Quantum games provide mathematical settings for exploring competing interactions, e.g. between classical and quantum agents [8,14,50], between players in the Prisoners' Dilemma game [9,10,40,46,72,73], multiplayer quantum games [12,19,28,29,38,39,43,56,62,74,75], interactions on quantum networks [53,54,63], to name a few. Moreover, a number of authors are using game-theoretic settings to explore the possibility of new quantum algorithms and protocols [8,53,76–78]. The area of quantum auctions [79–81] is an example of this, providing new motivation for quantum computational and quantum network hardware. Thus far, a number of simpler quantum games have been implemented in hardware, demonstrating future promise [20,33,39,44,60,82].

The speed with which new quantum technologies are emerging suggests that soon it would be usual to take full advantage of quantum theory and, using quantum strategies, to beat an opposing player at some realistic game that uses quantum technology [50]. Also, there are suggestions that quantum games can potentially provide new insights into the rise of complexity and self-organization at the molecular level where the rules are dictated by quantum mechanics [9,48].

In game theory, some of the simplest games to analyse are the bimatrix games. In the area of quantum games, a well-known quantization scheme for bimatrix games was proposed by Eisert *et al.* [9]. In this scheme, the players' strategies or actions are particular local unitary transformations performed on an initial maximally entangled state |*ψ*_*i*_〉 in 2⊗2 Hilbert space. After the players' actions, the quantum state passes through an unentangling gate and thereafter is called the final state |*ψ*_*f*_〉. This state is subsequently measured using Stern–Gerlach type detectors generating four quantum probabilities [4]. players' pay-off relations are expressed in terms of the pay-off entries of the corresponding bimatrix and the obtained quantum probabilities. Experimental realizations of quantum games are described elsewhere [20,44,60]. The measurement basis for the final state |*ψ*_*f*_〉 is defined [9,10] by associating pure classical strategies of the players with the four basis vectors of the two-qubit quantum state. players' pay-off relations contain four quantum probabilities obtained by projecting the final quantum state onto the basis vectors and are expressed in terms of players' local unitary transformations and the projection postulate of quantum mechanics.

The consideration of four quantum probabilities in the players' pay-off relations leads one to ask whether the pay-off relations in the quantum game can be described as mixed-strategy pay-off relations in the classical game. This is the case when quantum probabilities are factorizable and then one can express quantum probabilities in terms of players' mixed strategies. The relations describing factorizability of quantum probabilities are a set of equations that link players' mixed strategies to a probability distribution on players' pay-offs.

A set of quantum probabilities can be non-factorizable and thus cannot be obtained from the players' mixed strategies. A quantum game can then be described as the game in which non-factorizable probabilities are permitted. Approaching a quantum game from this perspective, this paper presents two approaches in obtaining games with non-factorizable probabilities. In our first approach, factorizability is controlled by an external parameter *k*∈[0,1] in the sense that assigning *k*=0 results in the factorizable game, whereas assigning *k*≠0 leads to a non-factorizable game. We then ask whether a general quantum probability distribution can be described in this way. In our second approach, we reexpress the players' pay-off relations in a form that allows us to obtain a non-factorizble game directly from the factorizable game by defining a function of players' strategies that satisfies certain constraints.

## 2. Two-player quantum games

Consider a symmetric bimatrix game [5–7]

2.1 $$\begin{array}{c} \text{Alice} \end{array}\;\begin{array}{c} S_{1}\\ S_{2} \end{array}\;\overset{\overset{\begin{array}{c} \text{Bob} \end{array}}{\begin{array}{ccc} S_{1}^{\prime } & & S_{2}^{\prime } \end{array}}}{\begin{array}{cc} \left(\alpha ,\alpha \right) & \left(\beta ,\gamma \right)\\ \left(\gamma ,\beta \right) & (\delta ,\delta ), \end{array}}$$

where *α*, *β*, *γ* and *δ* are real numbers. Assume that Alice's and Bob's mixed strategies are *p*,*q*∈[0,1], respectively, at which their pay-offs can be written as

2.2 $$\begin{array}{rl} \Pi_{A}(p,q) & =\alpha pq+\beta p(1-q)+\gamma (1-p)q+\delta (1-p)(1-q)\\ \text{and}\;\Pi_{B}(p,q) & =\alpha pq+\gamma p(1-q)+\beta (1-p)q+\delta (1-p)(1-q). \end{array}\}$$

A Nash equilibrium (NE) consists of the pair (p*,q*) of strategies such that no player has any motivation to unilaterally deviate from it. The game's Nash inequalities take the form

2.3 $$\Pi_{A}(p^{*},q^{*})-\Pi_{A}(p,q^{*})\geq 0\;\mathrm{and}\;\Pi_{B}(p^{*},q^{*})-\Pi_{B}(p^{*},q)\geq 0,$$

which take the following form for the game defined by matrix (2.1):

2.4 $$\begin{array}{rl} \left[(\alpha -\beta -\gamma +\delta )q^{*}+(\beta -\delta )](p^{*}-p\right) & \geq 0\\ \text{and}\;[(\alpha -\beta -\gamma +\delta )p^{*}+(\beta -\delta )](q^{*}-q) & \geq 0, \end{array}\}$$

that gives the classical mixed strategy description of the matrix game (2.1).

Now consider the game (2.1) when played in Eisert *et al.*'s quantization scheme [9,10]. The scheme uses two qubits to play a quantum version of the game (2.1), whose quantum state is in 2⊗2-dimensional Hilbert space. In view of the game (2.1), a measurement basis for quantum state of two qubits is chosen as $\left|S_{1}S_{1}^{\prime }\right\rangle$, $\left|S_{1}S_{2}^{\prime }\right\rangle$, $\left|S_{2}S_{1}^{\prime }\right\rangle$, $\left|S_{2}S_{2}^{\prime }\right\rangle$. An entangled initial quantum state |*ψ*_*i*_〉 is obtained by using a two-qubit entangling gate $\hat{J}$, i.e. $\left|\psi_{i}\rangle =\hat{J}|S_{1}S_{1}^{\prime }\right\rangle$, where $\hat{J}=\exp\{i\gamma S_{2}⊗S_{2}^{\prime }/2\}$ and *γ* ∈[0,*π*/2] is a measure of the game's entanglement. For a separable or product game *γ*=0, whereas for a maximally entangled game *γ*=*π*/2. The scheme considers the initial state being a maximally entangled state |*ψ*_*i*_〉. Players perform their local unitary transformations ${\hat{U}}_{A}$ and ${\hat{U}}_{B}$ from two sets of unitary transformations:

2.5 $$U(\theta )=\left(\begin{array}{cc} \cos\left(\frac{\theta }{2}\right) & \sin\left(\frac{\theta }{2}\right)\\ -\sin\left(\frac{\theta }{2}\right) & \cos\left(\frac{\theta }{2}\right) \end{array}\right)$$

and

2.6 $$U(\theta ,\phi )=\left(\begin{array}{cc} e^{i\phi }\cos\left(\frac{\theta }{2}\right) & \sin\left(\frac{\theta }{2}\right)\\ -\sin\left(\frac{\theta }{2}\right) & e^{-i\phi }\cos\left(\frac{\theta }{2}\right) \end{array}\right),$$

where 0≤*θ*≤*π* and 0≤*ϕ*≤*π*/2. These actions change the initial maximally entangled state |*ψ*_*i*_〉 to $\left({\hat{U}}_{A}⊗{\hat{U}}_{B})\hat{J}|S_{1}S_{1}^{\prime }\right\rangle$ which then is passed through an untangling gate ${\hat{J}}^{†}$ and the state of the game changes to the final state, i.e. $\left|\psi_{f}\rangle ={\hat{J}}^{†}({\hat{U}}_{A}⊗{\hat{U}}_{B})\hat{J}|S_{1}S_{1}^{\prime }\right\rangle$. The state |*ψ*_*f*_〉 is measured in the basis $\left|S_{1}S_{1}^{\prime }\right\rangle$, $\left|S_{1}S_{2}^{\prime }\right\rangle$, $\left|S_{2}S_{1}^{\prime }\right\rangle$, $\left|S_{2}S_{2}^{\prime }\right\rangle$ and using the quantum probability rule, the players' pay-offs are obtained as

2.7 $$\begin{array}{rl} \Pi_{A}({\hat{U}}_{A},{\hat{U}}_{B}) & =\alpha |\langle S_{1}S_{1}^{\prime }|\psi_{f}\rangle {|}^{2}+\beta |\langle S_{1}S_{2}^{\prime }|\psi_{f}\rangle {|}^{2}+\gamma |\langle S_{2}S_{1}^{\prime }|\psi_{f}\rangle {|}^{2}+\delta |\langle S_{2}S_{2}^{\prime }|\psi_{f}\rangle {|}^{2}\\ \text{and}\;\Pi_{B}({\hat{U}}_{A},{\hat{U}}_{B}) & =\alpha |\langle S_{1}S_{1}^{\prime }|\psi_{f}\rangle {|}^{2}+\gamma |\langle S_{1}S_{2}^{\prime }|\psi_{f}\rangle {|}^{2}+\beta |\langle S_{2}S_{1}^{\prime }|\psi_{f}\rangle {|}^{2}+\delta |\langle S_{2}S_{2}^{\prime }|\psi_{f}\rangle {|}^{2}. \end{array}\}$$

The NE for the quantum game consists of a pair $\left({\hat{U}}_{A}^{*},{\hat{U}}_{B}^{*}\right)$ of local unitary transformations that are obtained from the inequalities

2.8 $$\Pi_{A}({\hat{U}}_{A}^{*},{\hat{U}}_{B}^{*})-\Pi_{A}({\hat{U}}_{A},{\hat{U}}_{B}^{*})\geq 0\;\mathrm{and}\;\Pi_{B}({\hat{U}}_{A}^{*},{\hat{U}}_{B}^{*})-\Pi_{B}({\hat{U}}_{A}^{*},{\hat{U}}_{B})\geq 0.$$

That is, the NE consists a pair of two-parameter unitary transformations (2.6), corresponding to the two players, such that neither player is left with any motivation to deviate from it. For *α*=3, *β*=0, *γ*=5 and *δ*=1, the matrix (2.1) gives the game of Prisoners' Dilemma for which a Pareto-optimal NE is obtained as Eisert *et al*. [9]

2.9 $$\hat{Q}={\hat{U}}_{A}\left(0,\frac{\pi }{2}\right)={\hat{U}}_{B}\left(0,\frac{\pi }{2}\right)$$

at which the players' pay-offs are $\Pi_{A}(\hat{Q},\hat{Q})=\Pi_{B}(\hat{Q},\hat{Q})=3.$ Thus, the quantum strategy $\hat{Q}∼\hat{U}(0,\pi /2)$ emerges as the new equilibrium, when both players have access to the two-parameter set (2.6) of unitary 2⊗2 operators.

Benjamin & Hayden [13] observed that when their two-parameter set is extended to include all local unitary operations (i.e. all of *SU*(2)), the strategy $\hat{Q}$ does not remain an equilibrium. They showed that in the full space of deterministic quantum strategies there exists no equilibrium for the quantum Prisoners' Dilemma. Also, they observed that the set (2.6) of two-parameter quantum strategies is not closed under composition, although this closure is the necessary requirement for any set of quantum strategies. It can be explained as follows. Eisert *et al*. [9,10] permitted both players the same strategy set but introduced an arbitrary constraint into that set. This amounts to permitting a certain strategy while forbidding the logical counter strategy which one would intuitively expect to be equally allowed. Benjamin & Hayden showed [13] that $\hat{Q}$ emerges as the ideal strategy only because of restricting the strategy set arbitrarily.

Notwithstanding the above observations, we note that in the two-player quantum game, pairs of unitary transformations are players' strategies that are mapped to a set of four quantum probabilities that are normalized to 1 and in terms of which the players' pay-off relations are expressed. This mapping is achieved via the quantum probability rule [4] that obtains a quantum probability by squaring the modulus of the projections of the final quantum state to a basis state in the Hilbert space. This mapping re-expresses the four quantum probabilities in terms of the pair of players' unitary transformations. This re-expression opens up the route to finding Nash equilibria of the game as pairs of unitary transformations. In the following, we revise quantization of a two-player game, discuss a factorizable game and present the two approaches that lead to obtaining a non-factorizable game.

## 3. Factorizability of a set of quantum probabilities

We note that the pay-off relations (2.7) can be written as

3.1 $$\begin{array}{rl} \Pi_{A}({\hat{U}}_{A},{\hat{U}}_{B}) & =\alpha \epsilon_{1}+\beta \epsilon_{2}+\gamma \epsilon_{3}+\delta \epsilon_{4}\\ \text{and}\;\Pi_{B}({\hat{U}}_{A},{\hat{U}}_{B}) & =\alpha \epsilon_{1}+\gamma \epsilon_{2}+\beta \epsilon_{3}+\delta \epsilon_{4}, \end{array}\}$$

where

3.2 $$\begin{array}{rl} \epsilon_{1} & =|\langle S_{1}S_{1}^{\prime }|\psi_{f}\rangle {|}^{2},\;\epsilon_{2}=|\langle S_{1}S_{2}^{\prime }|\psi_{f}\rangle {|}^{2}\\ \text{and}\;\epsilon_{3} & =|\langle S_{2}S_{1}^{\prime }|\psi_{f}\rangle {|}^{2},\;\epsilon_{4}=|\langle S_{2}S_{2}^{\prime }|\psi_{f}\rangle {|}^{2} \end{array}\}$$

are four quantum probabilities obtained using the quantum probability rule, i.e. projecting the final quantum state |*ψ*_*f*_〉 of the game to the four basis vectors $\left|S_{1}S_{1}^{\prime }\right\rangle$, $\left|S_{1}S_{2}^{\prime }\right\rangle$, $\left|S_{2}S_{1}^{\prime }\right\rangle$, $\left|S_{2}S_{2}^{\prime }\right\rangle$. The probabilities *ϵ*_*i*_ are normalized, i.e.

3.3 $$\sum_{i=1}^{4}\epsilon_{i}=1.$$

Comparing the pay-off relations in the classical game (2.2) and in the quantum game (3.1), we note that the pay-off relations in the quantum game can be reduced to the pay-off relations in the classical game when probabilities *ϵ*_*i*_ are factorizable. Probabilities *ϵ*_*i*_ are factorizable when for a given set of values assigned in range [0,1] to probabilities *ϵ*_*i*_, we can find two probabilities *p*,*q*∈[0,1] such that *ϵ*_*i*_ can be written in terms of *p* and *q* as

3.4 $$\epsilon_{1}=pq,\;\epsilon_{2}=p(1-q),\;\epsilon_{3}=(1-p)q\;\mathrm{and}\;\epsilon_{4}=(1-p)(1-q).$$

That is, in the classical mixed-strategy version of the two-player game, the four probabilities *ϵ*_*i*_ appearing in the pay-off relations (2.2) are re-expressed in terms of players' strategic variables *p* and *q* via the factorizability conditions (3.4). If this is the case for the probabilities *ϵ*_*i*_, then we can associate the probabilities *p* and *q* to the players Alice and Bob, respectively, so that the pay-off relations in the quantum game (3.1) are interpreted as corresponding to a mixed-strategy classical game, i.e.

3.5 $$\Pi_{A}({\hat{U}}_{A},{\hat{U}}_{B})=\Pi_{A}(p,q)\;\mathrm{and}\;\Pi_{B}({\hat{U}}_{A},{\hat{U}}_{B})=\Pi_{B}(p,q),$$

with *p* and *q* satisfying the factorizability relations (3.4). In (3.4), *ϵ*_*i*_ represent not just a specific set of four numbers in [0,1] that add up to 1, but the entirety of such four numbers that can be generated by the quantum mechanical set-up used for playing a quantum game. As it can be seen from the equation (3.1), in Eisert *et al.*'s scheme, the full range of the players' pay-offs become accessible by giving players access to unitary transformations. In other words, a pair of unitary transformations results in a normalized set of four probabilities and Nash inequalities lead us to obtaining a pair of unitary transformations as an NE.

## 4. Games with non-factorizable probabilities

Quantum probabilities *ϵ*_*i*_, however, may not be factorizable in the sense described by equation (3.4). That is, the measurement stage of a quantum game can result in such probabilities *ϵ*_*i*_ (0≤*ϵ*_*i*_≤1) that one cannot find *p*,*q*∈[0,1] so that equations (3.4) are satisfied. Viewing the pay-off relations (3.1) from this probabilistic viewpoint, it then seems natural to ask whether we can obtain the pay-offs (3.1) by simply removing the factorizability relations (3.4), and if this is the case then what are the possibly new outcomes of the game. A quantum game allows obtaining sets of non-factorizable probabilities, and in this paper we look at the role of non-factorizable probabilities on the outcome of a game. This can also be stated as follows. Considering equations (3.1) and (3.5), we can describe the factorizable game as the one for which

4.1 $$\Pi_{A}(p,q)=\alpha \epsilon_{1}+\beta \epsilon_{2}+\gamma \epsilon_{3}+\delta \epsilon_{4}\;\mathrm{and}\;\Pi_{B}(p,q)=\alpha \epsilon_{1}+\gamma \epsilon_{2}+\beta \epsilon_{3}+\delta \epsilon_{4},$$

where $\Sigma_{i=1}^{4}\epsilon_{i}=1$ and the probabilities *ϵ*_*i*_ are related to players's strategies *p* and *q* via the factorizability relations (3.4). In the following, we consider two approaches in obtaining non-factorizable probabilities.

### 4.1 The first approach

Our first approach considers an external parameter *k* that determines whether the probabilities *ϵ*_*i*_ (0 ≤ *i* ≤ 4) are factorizable or not. For this, we consider the following probability distribution

4.2 $$\begin{array}{rl} \epsilon_{1} & ={\left(2k-1\right)}^{2}pq,\;\epsilon_{2}=(1-k)p(1-q)+kq(1-p)\\ \text{and}\;\epsilon_{3} & =(1-k)q(1-p)+kp(1-q),\;\epsilon_{4}=4k(1-k)pq+(1-p)(1-q). \end{array}\}$$

It can be confirmed that for *k* in the range [0,1], we have 0≤*ϵ*_*i*_≤1 and that probabilities *ϵ*_*i*_ are normalized according to the equation (3.3). When *k*=0, the distribution (4.2) reduces to the factorizable distribution of the equations (3.4). However, when *k* is non-zero, the probability distribution (4.2) is not factorizable. Using the pay-off relations (4.1), we now obtain the pay-offs for Alice and Bob for the distribution (4.2) as

4.3 $$\begin{array}{rl} \Pi_{A}(p,q,k) & =\alpha {\left(2k-1\right)}^{2}pq+\beta [(1-k)p(1-q)+kq(1-p)]\\ & \;+\gamma [(1-k)q(1-p)+kp(1-q)]+\delta [4k(1-k)pq+(1-p)(1-q)]\\ \text{and}\;\Pi_{B}(p,q,k) & =\alpha {\left(2k-1\right)}^{2}pq+\gamma [(1-k)p(1-q)+kq(1-p)]\\ & \;+\beta [(1-k)q(1-p)+kp(1-q)]+\delta [4k(1-k)pq+(1-p)(1-q)]. \end{array}\}$$

The NE strategy pair (p*,q*) is then obtained from the inequalities

4.4 $$\begin{array}{rl} \left\{[\alpha {\left(1-2k\right)}^{2}-\beta -\gamma +\delta \{1+4k(1-k)\}]q^{*}+(\beta -\delta )-k(\beta -\gamma )\}(p^{*}-p\right) & \geq 0\\ \text{and}\;\{[\alpha {\left(1-2k\right)}^{2}-\beta -\gamma +\delta \{1+4k(1-k)\}]p^{*}+(\beta -\delta )-k(\beta -\gamma )\}(q^{*}-q) & \geq 0. \end{array}\}$$

Now, for the Prisoners' Dilemma game considered above, we have *α*=3, *β*=0, *γ*=5 and *δ*=1 and these reduce the above NE conditions to

4.5 $$\begin{array}{rl} \left\{-1-q+k[5-8q(1-k)]\}(p^{*}-p\right) & \geq 0\\ \text{and}\;\{-1-p+k[5-8p(1-k)]\}(q^{*}-q) & \geq 0. \end{array}\}$$

For *k*=0, these relations give the NE in the classical factorizable game, i.e. (p*,q*)=(0,0) at which the players pay-offs are obtained as *Π*_A,*B*_(p*,q*,*k*)=*Π*_A,*B*_(0,0,0)=1.

Now consider the case when *k*=1 for which we find the NE being (p*,q*)=(1,1) and the players' pay-offs are obtained as *Π*_A,*B*_(*p*,*q*,*k*)=*Π*_A,*B*_(1,1,1)=3. These pay-offs are same as obtained in the maximally entangled quantum game in Eisert *et al.*'s scheme [9] with both players playing the quantum strategy $\hat{Q}$ defined in equation (2.9).

However, we note that the probability distribution in equation (4.2) is not as general as a quantum probability distribution can be within a quantum game. This is because for (4.2) to be a quantum probability distribution, it is required to obey only the normalization constraint (3.3). Although the probability distribution (4.2) is normalized, it also obeys other restrictions because of its particular form and the way it is defined. This can also be stated as follows. Whereas the probability distribution (4.2) is normalized, not every normalized quantum probability distribution will have this form. That is, there can be quantum probability distributions that cannot be written in the same form as the distribution (4.2). However, in spite of these limitations, the probability distribution (4.2) demonstrates the effect of a non-factorizable probability distribution on the outcome of a game. It also produces the classical game as a special case. In the following, we present a second approach that defines a probability distribution using a function of players' strategies *p* and *q* that is subject to certain constraints.

### 4.2 The second approach

At this stage, we note that when *ϵ*_*i*_ are factorizable in the sense described by equations (3.4) that gives the relationship between *p*, *q* and *ϵ*_*i*_. Using these relations, the players' strategies *p* and *q* can be expressed in terms of probabilities *ϵ*_*i*_ as

4.6 $$p=\epsilon_{1}+\epsilon_{2}\;\mathrm{and}\;q=\epsilon_{1}+\epsilon_{3},$$

and using equations (3.2), we can write equations (4.6) as

4.7 $$\begin{array}{rl} p & =|\langle S_{1}S_{1}^{\prime }|\psi_{f}\rangle {|}^{2}+|\langle S_{1}S_{2}^{\prime }|\psi_{f}\rangle {|}^{2}\\ \text{and}\; & =|\langle S_{1}S_{1}^{\prime }|\psi_{f}\rangle {|}^{2}+|\langle S_{2}S_{1}^{\prime }|\psi_{f}\rangle {|}^{2}. \end{array}\}$$

Knowing that *p* and *q* are the players' strategies, and each player has the freedom to play whatever she/he likes, the strategies *p* and *q* are considered to be independent of each other.

When quantum probabilities *ϵ*_*i*_ are factorizable in the sense described by equations (3.4), the pay-off relations (3.1) can also be interpreted in terms of playing a game that involves tossing a pair of coins as follows. Consider a pair of biased coins that are tossed together. Either coin can land in the head (H) or the tail (T) state and for the pair we can define

4.8 $$\epsilon_{1}=\mathrm{Pr}(H,H),\;\epsilon_{2}=\mathrm{Pr}(H,T),\;\epsilon_{3}=\mathrm{Pr}(T,H)\;\mathrm{and}\;\epsilon_{4}=\mathrm{Pr}(T,T)$$

as being the probabilities of the coins landing in the (*H*,*H*), (*H*,*T*), (*T*,*H*), (*T*,*T*) states, respectively. Here, for instance, $\epsilon_{2}=\mathrm{Pr}(H,T)$ is the probability that the first coin (or Alice's coin) lands in the H state whereas the second coin (or Bob's coin) lands in the T state. Now, referring to the equation (4.6), *p* can then be interpreted as being the probability, in the joint toss of two coins, that the first coin lands in the H state and, likewise, *q* can be interpreted as being the probability that the second coin lands in the H state.

However, we are interpreting *p* and *q* as being the players' strategies which means that a player plays his/her strategy by changing the bias of the coin to which he/she is given access to. Also, we note that, for factorizable quantum probabilities, using equations (3.4) and (4.6), we can write

4.9 $$\begin{array}{rl} \epsilon_{1} & =(\epsilon_{1}+\epsilon_{2})(\epsilon_{1}+\epsilon_{3}),\;\epsilon_{2}=(\epsilon_{1}+\epsilon_{2})(1-\epsilon_{1}-\epsilon_{3})\\ \text{and}\;\epsilon_{3} & =(1-\epsilon_{1}-\epsilon_{2})(\epsilon_{1}+\epsilon_{3}),\;\epsilon_{4}=(1-\epsilon_{1}-\epsilon_{2})(1-\epsilon_{1}-\epsilon_{3}). \end{array}\}$$

We now suggest another approach in considering the players' pay-offs (3.1) in the quantum game. In view of the relations *p*=*ϵ*_1_+*ϵ*_2_ and *q*=*ϵ*_1_+*ϵ*_3_, and the normalization constraint $\Sigma_{i=1}^{4}\epsilon_{i}=1,$ we can rewrite the pay-offs (3.1) as

4.10 $$\begin{array}{rl} \Pi_{A}({\hat{U}}_{A},{\hat{U}}_{B}) & =\alpha \epsilon_{1}+\beta \epsilon_{2}+\gamma \epsilon_{3}+\delta \epsilon_{4}\\ & =\epsilon_{1}(\alpha -\beta -\gamma +\delta )+(\beta -\delta )(\epsilon_{1}+\epsilon_{2})+(\gamma -\delta )(\epsilon_{1}+\epsilon_{3})+\delta \\ & =\epsilon_{1}(\alpha -\beta -\gamma +\delta )+(\beta -\delta )p+(\gamma -\delta )q+\delta \\ \text{and}\;\Pi_{B}({\hat{U}}_{A},{\hat{U}}_{B}) & =\alpha \epsilon_{1}+\gamma \epsilon_{2}+\beta \epsilon_{3}+\delta \epsilon_{4}\\ & =\epsilon_{1}(\alpha -\beta -\gamma +\delta )+(\gamma -\delta )p+(\beta -\delta )q+\delta . \end{array}\}$$

We also note that the players' pay-offs (2.2) in the factorizable game can be expressed as

4.11 $$\begin{array}{rl} \Pi_{A}(p,q) & =\alpha pq+\beta p(1-q)+\gamma (1-p)q+\delta (1-p)(1-q)\\ & =pq(\alpha -\beta -\gamma +\delta )+(\beta -\delta )p+(\gamma -\delta )q+\delta \\ \text{and}\;\Pi_{B}(p,q) & =\alpha pq+\gamma p(1-q)+\beta (1-p)q+\delta (1-p)(1-q)\\ & =pq(\alpha -\beta -\gamma +\delta )+(\gamma -\delta )p+(\beta -\delta )q+\delta . \end{array}\}$$

Comparing equations (4.11) and (4.10), we note that taking *ϵ*_1_=*pq* makes the pay-offs (4.10) equivalent to the pay-offs (4.11) in the factorizable game. That is, with players' strategic variables being *p*=*ϵ*_1_+*ϵ*_2_ and *q*=*ϵ*_1_+*ϵ*_3_ and *ϵ*_1_=*pq*, the players' pay-offs in the quantum game are reduced to their pay-offs in the factorizable game. Also, the product *ϵ*_1_=*pq* takes the pair (*p*,*q*) to the interval [0,1] and is responsible for reducing the right-hand sides of equations (4.10) to the right-hand sides of equations (4.11).

This motivates considering *ϵ*_1_ as a function of the players' independent strategic variables *p* and *q*, i.e. *ϵ*_1_=*ϵ*_1_(*p*,*q*). It is a function of the players' strategic variables *p* and *q* that maps the pair (*p*,*q*) to the interval [0,1]. Taking *ϵ*_1_=*ϵ*_1_(*p*,*q*) allows us to consider forms of the function *ϵ*_1_(*p*,*q*) that are different from the product *pq*. In turn, this permits departing from the factorizability relations (3.4) and thus from the factorizable game.

We also note from equations (4.10) that the function *ϵ*_1_ connects the two players' pay-offs together and reminds us of the term *γ* representing the measure of entanglement in the players' pay-off relations within the quantum game that is played in Eisert *et al*.'s scheme. We write the pay-off relations (4.10) for a non-factorizable game as

4.12 $$\begin{array}{rl} \Pi_{A}(p,q) & =\epsilon_{1}(p,q)(\alpha -\beta -\gamma +\delta )+(\beta -\delta )p+(\gamma -\delta )q+\delta \\ \text{and}\;\Pi_{B}(p,q) & =\epsilon_{1}(p,q)(\alpha -\beta -\gamma +\delta )+(\gamma -\delta )p+(\beta -\delta )q+\delta \end{array}\}$$

and consider it being the defining pay-off relations for the non-factorizable game. Note that for the quantum game the players' strategies are unitary transformations ${\hat{U}}_{A}$ and ${\hat{U}}_{B}$, whereas for the new game defined by pay-off relations (4.12) the players' strategies are *p*,*q*∈[0,1]. Also, the two players' pay-offs are linked together via the function *ϵ*_1_=*ϵ*_1_(*p*,*q*). The game defined by the pay-off relations (4.12) makes no reference to the quantum operations and can thus simply be called a non-factorizable game or a game that permits non-factorizable probabilities.

With equations (4.12) being the defining pay-off relations for our non-factorizable game, an immediate question would be what are the restrictions on the type of functions *ϵ*_1_(*p*,*q*). To determine this, we note that the permissible ranges of the players' pay-offs in equations (4.12) and (3.1) must be identical. In view of the right-hand sides of equations (3.1), we put equations (4.12) in the following form

4.13 $$\begin{array}{rl} \Pi_{A}(p,q) & =\alpha \epsilon_{1}(p,q)+\beta [p-\epsilon_{1}(p,q)]+\gamma [q-\epsilon_{1}(p,q)]+\delta [\epsilon_{1}(p,q)-(p+q)+1]\\ \text{and}\;\Pi_{B}(p,q) & =\alpha \epsilon_{1}(p,q)+\gamma [p-\epsilon_{1}(p,q)]+\beta [q-\epsilon_{1}(p,q)]+\delta [\epsilon_{1}(p,q)-(p+q)+1] \end{array}\}$$

and for the right-hand sides of these equations to be identical to the right-hand sides of equations (3.1), we require

4.14 $$\epsilon_{1}(p,q)\leq p,\;\epsilon_{1}(p,q)\leq q\;\mathrm{and}\;\epsilon_{1}(p,q)\leq p+q.$$

Note that the right-hand sides of the pay-off relations (4.13) are equivalent to the right-hand sides of the pay-off relations (3.1) in the quantum game. This can be confirmed as follows. Referring to equations (4.12), we note that for given *p*,*q*∈[0,1] as players' independent strategies, the function *ϵ*_1_(*p*,*q*) gives a value in [0,1], the functions *ϵ*_2_(*p*,*q*) and *ϵ*_3_(*p*,*q*) can then be defined as

4.15 $$\begin{array}{rl} \epsilon_{2}(p,q) & =p-\epsilon_{1}(p,q),\;\epsilon_{3}(p,q)=q-\epsilon_{1}(p,q)\\ \text{and}\;\epsilon_{4}(p,q) & =1-[(p+q)-\epsilon_{1}(p,q)]. \end{array}\}$$

When the restrictions (4.14) hold, the functions *ϵ*_2_(*p*,*q*), *ϵ*_3_(*p*,*q*) and *ϵ*_4_(*p*,*q*) produce values within the range [0,1]. Of course, the function *ϵ*_1_(*p*,*q*)=*pq*, which results in the factorizable game, satisfies these requirements. In view of the restrictions (4.14), and a non-factorizable game for which *ϵ*_1_(*p*,*q*)≠*pq*, we require the function *ϵ*_1_(*p*,*q*) to be restricted by the following condition:

4.16 $$\epsilon_{1}(p,q)\leq pq.$$

Examples of the functions that satisfy this requirement include *ϵ*_1_(*p*,*q*)=(*pq*)^2^ and *ϵ*_1_(*p*,*q*)=*p*^2^*q*^3^, among others.

Note that, in view of (4.14), the range of function *ϵ*_1_(*p*,*q*) and the values assigned to *p* and *q*, as being players' strategies, are in the interval [0,1]. In this case, equations (4.15) generate values for *ϵ*_2_(*p*,*q*), *ϵ*_3_(*p*,*q*) and *ϵ*_4_(*p*,*q*) in the range [0,1]. Also, equations (4.15) show that $\sum_{i=1}^{4}\epsilon_{i}(p,q)=1$, and thus *ϵ*_*i*_(*p*,*q*) are probabilities for 1≤*i*≤4. The converse is also true. That is, for given values for four normalized probabilities *ϵ*_*i*_ (1≤*i*≤4), using equation (4.6) we can determine values for *p* and *q* as being *p*=*ϵ*_1_+*ϵ*_2_ and *q*=*ϵ*_1_+*ϵ*_3_.

#### 4.2.1 Nash equilibria for the game with non-factorizable probabilities

The pair of strategies (p*,q*) defining Nash equilibria are obtained from the inequalities

4.17 $$\Pi_{A}(p^{*},q^{*})-\Pi_{A}(p,q^{*})\geq 0\;\mathrm{and}\;\Pi_{B}(p^{*},q^{*})-\Pi_{B}(p^{*},q)\geq 0,$$

which, for the game (4.12), can be written as

4.18 $$\begin{array}{rl} \Pi_{A}(p^{*},q^{*})-\Pi_{A}(p,q^{*}) & =[\epsilon_{1}(p^{*},q^{*})-\epsilon_{1}(p,q^{*})](\alpha -\beta -\gamma +\delta )+(p^{*}-p)(\beta -\delta )\geq 0\\ \text{and}\;\Pi_{B}(p^{*},q^{*})-\Pi_{B}(p^{*},q) & =[\epsilon_{1}(p^{*},q^{*})-\epsilon_{1}(p^{*},q)](\alpha -\beta -\gamma +\delta )+(q^{*}-q)(\beta -\delta )\geq 0. \end{array}\}$$

For *ϵ*_1_(*p*,*q*)=*pq* these equations are reduced to equations (2.4). For *ϵ*_1_(*p*,*q*)=*p*^2^*q*^2^, the inequalities (4.18) give

$$\begin{array}{rl} \Pi_{A}(p^{*},q^{*})-\Pi_{A}(p,q^{*}) & =(p^{*}-p)[q^{*2}(p^{*}+p)(\alpha -\beta -\gamma +\delta )+(\beta -\delta )]\geq 0\\ \mathrm{and}\;\Pi_{B}(p^{*},q^{*})-\Pi_{B}(p^{*},q) & =(q^{*}-q)[p^{*2}(q^{*}+q)(\alpha -\beta -\gamma +\delta )+(\beta -\delta )]\geq 0, \end{array}$$

and, likewise, for *ϵ*_1_(*p*,*q*)=*p*^2^*q*^3^ the inequalities (4.18) give

4.19 $$\begin{array}{rl} \Pi_{A}(p^{*},q^{*})-\Pi_{A}(p,q^{*}) & =(p^{*}-p)[q^{*3}(p^{*}+p)(\alpha -\beta -\gamma +\delta )+(\beta -\delta )]\geq 0\\ \text{and}\;\Pi_{B}(p^{*},q^{*})-\Pi_{B}(p^{*},q) & =(q^{*}-q)[p^{*2}(q^{*2}+qq^{*}+q^{2})(\alpha -\beta -\gamma +\delta )+(\beta -\delta )]\geq 0. \end{array}\}$$

Now consider the situation when the strategy pair (p*,q*)=(1,1) exists as an NE. As it is apparent from equations (2.4) that for *ϵ*_1_(*p*,*q*)=*pq*, the pair (p*,q*)=(1,1) exists as NE when (*α*−*γ*)≥0. For *ϵ*_1_(*p*,*q*)=*p*^2^*q*^2^ and the pair (p*,q*)=(1,1), we obtain

4.20 $$\begin{array}{rl} \Pi_{A}(1,1)-\Pi_{A}(p,1) & =(1-p)[(\alpha -\gamma )(1+p)+p(-\beta +\delta )]\geq 0\\ \text{and}\;\Pi_{B}(1,1)-\Pi_{B}(1,q) & =(1-q)[(\alpha -\gamma )(1+q)+q(-\beta +\delta )]\geq 0 \end{array}\}$$

and thus (p*,q*)=(1,1) exists as an NE when, in addition to (*α*−*γ*)≥0, we also have (*δ*−*β*)≥0. For *ϵ*_1_(*p*,*q*)=*p*^2^*q*^3^ and the same pair (p*,q*)=(1,1), we obtain

4.21 $$\begin{array}{rl} \Pi_{A}(1,1)-\Pi_{A}(p,1) & =(1-p)[(\alpha -\gamma )+p(\alpha -\beta -\gamma +\delta )]\geq 0\\ \text{and}\;\Pi_{B}(1,1)-\Pi_{B}(1,q) & =(1-q)[(\alpha -\gamma )+(q+q^{2})(\alpha -\beta -\gamma +\delta )]\geq 0. \end{array}\}$$

That is, when both (*α*−*γ*)≥0 and (*δ*−*β*)≥0 are true, we have the strategy pair (p*,q*)=(1,1) existing as an NE for both the cases, i.e. when *ϵ*_1_(*p*,*q*)=*p*^2^*q*^2^ and *p*^2^*q*^3^.

In this approach in extending a game from its classical mixed-strategy version to a non-classical version, the players' strategies involve one parameter for each, i.e. *p* and *q*∈[0,1]. We now refer to Eisert *et al.*'s quantum version of the same game [9,10] in which players' pay-off relations in the quantized version of the game involve one- and two-parameter strategy sets (2.5) and (2.6). As in our non-classical extension of a classical game the players have one-parameter strategy sets, it is appropriate to compare our non-classical extension above to the quantum version of Eisert *et al*. [9,10], when it involves one-parameter strategy sets. To achieve this, and with reference to the game (2.1), we recast the quantum game of Eisert *et al.* with one-parameter strategy set in the form of the non-classical game above. From Eisert *et al*. [10], we note that the players' pay-offs for one-parameter strategy set are

4.22 $$\begin{array}{rl} \Pi_{A}(\theta_{A},\theta_{B}) & =\alpha {\left|\cos\left(\frac{\theta_{A}}{2}\right)\cos\left(\frac{\theta_{B}}{2}\right)\right|}^{2}+\beta {\left|\sin\left(\frac{\theta_{B}}{2}\right)\cos\left(\frac{\theta_{A}}{2}\right)\right|}^{2}+\gamma {\left|\cos\left(\frac{\theta_{B}}{2}\right)\sin\left(\frac{\theta_{A}}{2}\right)\right|}^{2}\\ & \;+\delta {\left|\sin\left(\frac{\theta_{A}}{2}\right)\sin\left(\frac{\theta_{B}}{2}\right)\right|}^{2} \end{array}$$

and

4.23 $$\begin{array}{rl} \Pi_{A}(\theta_{A},\theta_{B}) & =\alpha {\left|\cos\left(\frac{\theta_{A}}{2}\right)\cos\left(\frac{\theta_{B}}{2}\right)\right|}^{2}+\gamma {\left|\sin\left(\frac{\theta_{B}}{2}\right)\cos\left(\frac{\theta_{A}}{2}\right)\right|}^{2}+\beta {\left|\cos\left(\frac{\theta_{B}}{2}\right)\sin\left(\frac{\theta_{A}}{2}\right)\right|}^{2}\\ & \;+\delta {\left|\sin\left(\frac{\theta_{A}}{2}\right)\sin\left(\frac{\theta_{B}}{2}\right)\right|}^{2}. \end{array}$$

Comparing equations (4.13) with equations (4.22), (4.23) and noting that 0≤*θ*≤*π*, whereas *p*, *q*∈[0,1], we define *p*=*θ*_A_/*π*, *q*=*θ*_B_/*π* and

$$\begin{array}{rl} \epsilon_{1}(p,q) & ={\left|\cos\left(\frac{\theta_{A}}{2}\right)\cos\left(\frac{\theta_{B}}{2}\right)\right|}^{2},\;p-\epsilon_{1}(p,q)={\left|\sin\left(\frac{\theta_{B}}{2}\right)\cos\left(\frac{\theta_{A}}{2}\right)\right|}^{2},\\ q-\epsilon_{1}(p,q) & ={\left|\cos\left(\frac{\theta_{B}}{2}\right)\sin\left(\frac{\theta_{A}}{2}\right)\right|}^{2},\;\epsilon_{1}(p,q)-(p+q)+1={\left|\sin\left(\frac{\theta_{A}}{2}\right)\sin\left(\frac{\theta_{B}}{2}\right)\right|}^{2}, \end{array}$$

from which one obtains

4.24 $$p=\cos^{2}\left(\frac{\theta_{A}}{2}\right),\;q=\cos^{2}\left(\frac{\theta_{B}}{2}\right),\;\epsilon_{1}(p,q)=pq$$

and that gives us the factorizable game as discussed just before the equation (4.12). This shows that Eisert *et al.*'s quantum game with one-parameter strategy set is identical to the classical factorizable game. For the same quantum game with two-parameter strategy set (2.6), the players' pay-offs are obtained as

4.25 $$\begin{array}{rl} \Pi_{A}(\theta_{A},\phi_{A};\theta_{B},\phi_{B}) & =\alpha {\left|\cos(\phi_{A}+\phi_{B})\cos\left(\frac{\theta_{A}}{2}\right)\cos\left(\frac{\theta_{B}}{2}\right)\right|}^{2}\\ & \;+\beta {\left|\cos(\phi_{A})\cos\left(\frac{\theta_{A}}{2}\right)\sin\left(\frac{\theta_{B}}{2}\right)-\sin(\phi_{B})\sin\left(\frac{\theta_{A}}{2}\right)\cos\left(\frac{\theta_{B}}{2}\right)\right|}^{2}\\ & \;+\gamma {\left|\sin(\phi_{A})\cos\left(\frac{\theta_{A}}{2}\right)\sin\left(\frac{\theta_{B}}{2}\right)-\cos(\phi_{B})\sin\left(\frac{\theta_{A}}{2}\right)\cos\left(\frac{\theta_{B}}{2}\right)\right|}^{2}\\ & \;+\delta {\left|\sin(\phi_{A}+\phi_{B})\cos\left(\frac{\theta_{A}}{2}\right)\cos\left(\frac{\theta_{B}}{2}\right)+\sin\left(\frac{\theta_{A}}{2}\right)\sin\left(\frac{\theta_{B}}{2}\right)\right|}^{2}. \end{array}$$

As was the case for one-parameter strategy set, we now compare equations (4.13) with equations (4.25) and (4.23). Recall that 0≤*θ*≤*π* and 0≤*ϕ*≤*π*/2, whereas *p*, *q*∈[0,1] to obtain

4.26 $$\begin{array}{rl} \epsilon_{1}(p,q) & ={\left|\cos(\phi_{A}+\phi_{B})\cos\left(\frac{\theta_{A}}{2}\right)\cos\left(\frac{\theta_{B}}{2}\right)\right|}^{2},\\ p-\epsilon_{1}(p,q) & ={\left|\cos(\phi_{A})\cos\left(\frac{\theta_{A}}{2}\right)\sin\left(\frac{\theta_{B}}{2}\right)-\sin(\phi_{B})\sin\left(\frac{\theta_{A}}{2}\right)\cos\left(\frac{\theta_{B}}{2}\right)\right|}^{2},\\ q-\epsilon_{1}(p,q) & ={\left|\sin(\phi_{A})\cos\left(\frac{\theta_{A}}{2}\right)\sin\left(\frac{\theta_{B}}{2}\right)-\cos(\phi_{B})\sin\left(\frac{\theta_{A}}{2}\right)\cos\left(\frac{\theta_{B}}{2}\right)\right|}^{2}\\ \text{and}\;\epsilon_{1}(p,q)-(p+q)+1 & ={\left|\sin(\phi_{A}+\phi_{B})\cos\left(\frac{\theta_{A}}{2}\right)\cos\left(\frac{\theta_{B}}{2}\right)+\sin\left(\frac{\theta_{A}}{2}\right)\sin\left(\frac{\theta_{B}}{2}\right)\right|}^{2}, \end{array}\}$$

and using these equations, *p* and *q* can then be expressed in terms of *θ*_A_,*ϕ*_A_,*θ*_B_,*ϕ*_B_, i.e.

4.27 $$p=p(\theta_{A},\phi_{A};\theta_{B},\phi_{B})\;\mathrm{and}\;q=q(\theta_{A},\phi_{A};\theta_{B},\phi_{B}).$$

That is, the two-parameter pay-off relations can be expressed in the form of (4.13) but this comes at the price that the players' strategic variables *p* and *q* are not local anymore. For the quantum game with a one-parameter set of strategies, this is indeed the case as we have *p*=*p*(*θ*_A_,*ϕ*_A_) and *q*=*q*(*θ*_A_,*ϕ*_A_).

## 5. Conclusion

We suggest a direct route to obtaining a non-factorizable game from a classical factorizable game while taking into consideration the quantum probabilities and the players' strategic variables. We explore how a quantum game can be considered as a non-factorizable game by considering the scheme of Eisert *et al.* for quantization of a bimatrix game that involves four quantum probabilities. When these probabilities are factorizable in a specific sense, as described by equations (3.4), the quantum game attains the interpretation of a mixed-strategy classical game. This paper presents two approaches in obtaining bimatrix games with non-factorizable probabilities. Our first approach discusses a non-factorizable probability distribution in which the non-factorizability is controlled by an external parameter *k*.

We note that the relations *p*=*ϵ*_1_+*ϵ*_2_ and *q*=*ϵ*_1_+*ϵ*_3_ as obtained from the factorizability constraints (3.4) are apparently the simplest expressions connecting the players' strategies *p* and *q* to the quantum probabilities *ϵ*_*i*_ (1≤*i*≤4). However, this does not mean that these are the only possible expressions that are consistent with the factorizability constraints (3.4). There can be other possible cases, for instance, when *p*=*p*(*ϵ*_*i*_) and *q*=*q*(*ϵ*_*i*_), i.e. both *p* and *q* are functions of all four quantum probabilities *ϵ*_*i*_ (1≤*i*≤4). In such a case, the factorizability constraints will take the following form

5.1 $$\begin{array}{rl} \epsilon_{1} & =p(\epsilon_{i})q(\epsilon_{i}),\;\epsilon_{2}=p(\epsilon_{i})[1-q(\epsilon_{i})],\;\epsilon_{3}=[1-p(\epsilon_{i})]q(\epsilon_{i})\\ \text{and}\;\epsilon_{4} & =[1-p(\epsilon_{i})][1-q(\epsilon_{i})] \end{array}\}$$

and, depending on how the functions *p*=*p*(*ϵ*_*i*_) and *q*=*q*(*ϵ*_*i*_) are defined, the analysis will lead to a different outcome of the quantum game. Essentially, in this paper we use the factorizability constraints (3.4) to obtain such functions that express players' strategies in terms of the probabilities *ϵ*_*i*_; there can be more than one possible way of doing that and each way has to respect the requirement that when *ϵ*_*i*_ are factorizable, now in the sense of equations (5.1), the quantum game reduces itself to the classical mixed-strategy game.

This paper addresses several questions relevant to the quantization scheme proposed by Eisert *et al*. in 1999. This scheme motivated other quantization schemes and has been the basis of a large number of research articles that have followed since then along this line of research. By systematically discussing the structure of the pay-off relations obtained in this scheme, and its relation to the corresponding classical mixed-strategy factorizable game, this paper presents an understanding of the role of quantum probabilities, factorizability of a probability distribution, and the nature of the players' strategic variables. We provide a perspective by which a non-factorizble game is obtained directly from a classical factorizable game by defining a function *ϵ*_1_(*p*,*q*) that satisfyies certain constraints. The quantum game of Eisert *et al*. involving one parameter strategy set is explained as the classical factorizable game. We then study functions *ϵ*_1_(*p*,*q*)=(*pq*)^2^ and *ϵ*_1_(*p*,*q*)=*p*^2^*q*^3^ that satisfy these constraints and lead to non-factorizable games. We determine the corresponding Nash equlibria. We then ask whether the quantum game with two-parameter set of strategies can be considered as a non-factorizable game for a particular choice of the function *ϵ*_1_(*p*,*q*). We find that this can be achieved but it comes at a significant cost that the players' strategic variables *p* and *q* do not remain local anymore. This is indeed the case for the quantum game with one-parameter set of strategies for which the players' strategic variables *p* and *q* are definable in terms of local variables, i.e. *p*=*p*(*θ*_A_,*ϕ*_A_) and *q*=*q*(*θ*_A_,*ϕ*_A_).
